# Use of telemonitoring in patient self-management of chronic disease: a qualitative meta-synthesis

**DOI:** 10.1186/s12872-023-03486-3

**Published:** 2023-09-19

**Authors:** Anna Creber, Donato Giuseppe Leo, Benjamin J. R. Buckley, Mahin Chowdhury, Stephanie L. Harrison, Masoud Isanejad, Deirdre A. Lane, on behalf of the TAILOR investigators

**Affiliations:** 1 School of Medicine, Faculty of Health and Life Sciences, University of Liverpool, Liverpool, UK; 2 Department of Cardiovascular and Metabolic Medicine, Institute of Life Course and Medical Sciences, Faculty of Health and Life Sciences, University of Liverpool, Liverpool, UK; 3 Liverpool Centre for Cardiovascular Science, University of Liverpool and Liverpool Heart & Chest Hospital, Liverpool, UK; 4 School of Sport and Exercise Sciences, Faculty of Science, Liverpool John Moores University, Liverpool, UK; 5 Registry of Senior Australians, South Australian Health and Medical Research Institute, Adelaide, Australia; 6 Department of Musculoskeletal Ageing, Institute of Life Course and Medical Sciences, Faculty of Health and Life Sciences, University of Liverpool, Liverpool, UK; 7 Department of Clinical Medicine, Aalborg University, Aalborg, Denmark

**Keywords:** Chronic disease, e-health, Meta-synthesis, Telehealth, Telemedicine, Telemonitoring

## Abstract

**Background:**

Telemonitoring for the remote patient self-management of chronic conditions can be a cost-effective method for delivering care in chronic disease; nonetheless, its implementation in clinical practice remains low. The aim of this meta-synthesis is to explore barriers and facilitators associated with the use of remote patient monitoring of chronic disease, drawing on qualitative research, and assessing participant interactions with this technology.

**Method:**

A meta-synthesis of qualitative studies was performed. MEDLINE, SCOPUS and the Cochrane Central Register of Controlled Trials (CENTRAL) were searched from database date of inception to 5 February 2021. The Critical Appraisal Skills Programme (CASP) was used to critically appraise each study. Thematic synthesis was performed to identify user (patients, carers and healthcare professionals) perspectives and experiences of patient remote monitoring of chronic disease (Type 2 diabetes mellitus, chronic obstructive pulmonary disease, and cardiovascular disease).

**Results:**

Searches returned 10,401 studies and following independent screening by two reviewers, nine studies were included in this meta-synthesis. Data were synthesised and categorised into four key themes: (1) *Improved care*; (2) *Communication*; (3) *Technology feasibility & acceptability*; and (4) *Intervention concerns*. Most patients using patient remote devices felt motivated in managing their own lifestyles and felt reassured by the close monitoring and increased communication. Barriers identified involved generational differences and difficulties with the technology used.

**Conclusion:**

Most studies showed a positive attitude to telemonitoring, with patients preferring the convenience of telemonitoring in comparison to attending regular clinics. Further research is required to assess the most effective technology for chronic disease management, how to maintain long-term patient adherence, and identify effective approaches to address generational variation in telemonitoring up-take.

**Supplementary Information:**

The online version contains supplementary material available at 10.1186/s12872-023-03486-3.

## Introduction

Globally, cardiovascular disease remains the leading cause of death among people with chronic disease, closely followed by chronic obstructive pulmonary disease (COPD). [1, 2] Fifteen million people in the UK have ≥ 1 long-term condition, [1, 3] with 70% of primary and acute care budgets spent on the management of these populations. [1] Due to the rapidly rising incidence of chronic diseases, effective management of these health condition has become imperative.

Telemonitoring is defined as the remote self-management of patients with chronic disease using telecommunication technology, enabling rapid and accurate information exchange between healthcare professionals (HCPs) and patients. [4] Telemonitoring can be an acceptable and cost-effective method for delivering effective care for people with chronic diseases. [4] Those experiencing chronic health conditions generally require ongoing medical attention with regular clinical visits, factors that can be mitigated by the implementation of telemonitoring with the advantages of saving patient and HCPs time, improving communication, and potentially reducing hospital admissions and National Health System (NHS) costs. [5–7].

Telemonitoring implementation within the UK has been slow, but it has increased during the Coronavirus disease (COVID-19) pandemic. [3, 8] The impact of COVID-19 has imposed additional healthcare challenges, resulting in rapid telemonitoring implementation and patients having to adapt to this change. [5] Broader barriers for patients include unfamiliarity and difficulties in technology access due to generational differences (where older patients experiences more difficulties in interacting with new technologies) and poor digital literacy, [9] and poor internet connectivity. [10] From the HCPs point of view, barriers toward this technology focus on the concerns with data protection, and on worries that telemonitoring may disrupt patient-doctor interaction. [11] Few meta-syntheses evaluating patients’ satisfaction, acceptability (how well the intervention is received by the target population), and feasibility (if the intervention is appropriate for future testing/implementation) of telemonitoring are present in the literature. [12–14] Existing research has focused on individual health conditions, such as COPD [12] and chronic musculoskeletal pain, [14] with none assessing the role of telemonitoring in patients with a broader range of long-term conditions and multimorbidity. Therefore, the aim of this review was to explore patients’ barriers and facilitators to, as well as HCPs considerations of, telemonitoring implementation in chronic disease self-management, drawing on available qualitative research and assessing participants interaction with this technology.

## Methods

This meta-synthesis complements a recently published systematic review and meta-analysis, [15] which evaluated the effectiveness of telemonitoring in the self-management of patients with chronic health conditions. The review was registered on the International Prospective Register of Systematic Reviews – PROSPERO (CRD42021236291) in accordance with the Preferred Reporting Items for Systematic Reviews and Meta-analyses (PRISMA) guidelines. [16].

### Inclusion Criteria

Studies that included qualitative components conducted in any setting and that explored the view on telemonitoring usage of patients aged 18 years and over and affected by at least one chronic condition among the following diseases: COPD, cardiovascular disease, and/or diabetes mellitus, were eligible for inclusion. In addition, HCPs views and considerations on the telemonitoring intervention were also included if available in the eligible papers.

### Participants

Adults (aged 18 years and over) were eligible for this review if reporting one or more of the following diseases: COPD, cardiovascular disease, and/or diabetes mellitus. HCPs views and considerations were also reported if available in the included papers.

### Intervention

Interventions involving remote collection of health information from patients with the use of digital technologies and the electronic transfer of this information to healthcare professionals for monitoring and assessment were eligible for inclusion in this review. Interventions were considered eligible only where a digital device for remote monitoring was provided, with the participant (or caregivers) taking physiological measurements (either manually and then inputting to the device or automatically uploaded by the measurement device). To be eligible for this review, the device had to transmit collected data to the participant’s healthcare team, which had to monitor the information and revise the treatment where required. Two-way exchange of information was required for the study to be included. We deemed this a remote patient management intervention.

### Comparator

Studies that compared a remote patient management intervention to usual care, other interventions or did not include a control group were considered eligible for this review.

### Outcomes

All studies that reported qualitative data looking at patients’ experience of telemonitoring were considered eligible for this review. HCPs experience was also considered were available.

### Search strategy

The search strategy was developed by the authors, who agreed on the key search terms. Databases searched included MEDLINE, SCOPUS and the Cochrane Central Register of Controlled Trials (CENTRAL). Relevant medical subject headings (MesH) terms and synonyms were used as search-terms including: ‘Telemedicine’, ‘Telehealth’, ‘Cardiovascular disease’, ‘Chronic obstructive pulmonary disease’, etc. and combined with Boolean operators, proximity operators, truncations, and wildcards (Supplementary Table 1). The search strategy used for this meta-synthesis is the same as that used in the previously published systematic review and meta-analysis. [15].

### Study screening

After removal of duplicates, selected study titles and abstracts were independently screened by two reviewers (DGL, MC) against agreed inclusion criteria. Additional study screening was independently undertaken by three other reviewers. Full text for each relevant study was retrieved and independently assessed for eligibility criteria. Discrepancies were resolved through consultation/review with the senior author (DAL).

### Quality assessment

Two researchers (AC, DGL) independently assessed study validity and credibility using the Critical Appraisal Skills Programme (CASP) qualitative checklist tool. [17] The CASP analysis tool assessed 10 areas: aim, methodology, research design, ethical considerations, research self-scrutiny, recruitment strategy, data-collection, data-analysis, findings, and study relevance. These were critically appraised using three primary measurements: ‘Yes’, ‘Can’t Tell’ and ‘No’. Discrepancies were discussed between researchers and those unresolved were addressed by the senior author (DAL).

### Data extraction

One researcher (AC) conducted data extraction, with key information extracted: (i) title, author, year, country, reference; (ii) study aim; (iii) study characteristics (sample size, study design, data analysis); (iv) participant characteristics (age, sex), (v) health condition; (vi) intervention description; (vii) qualitative data themes/ sub-themes. Data extraction for the intervention description included: (i) type of digital device provided; (ii) person taking the physiological measurement (patient or caregiver); (iii) type of data transmission (e.g., via internet, by text); (iv) staff monitoring the data transmitted (e.g., nurses, GPs); (v) methods of contacts with the patients (e.g., by phone, by text).

### Data Synthesis

Data synthesis was conducted using a manually-employed reflexive thematic approach. [18] One researcher read and re-read the studies. After undertaking initial quality assessment and data extraction; study results were synthesised and categorised into main themes and sub-themes. These were derived from identifying recurrent themes, with data classification by theme/sub-theme. These classifications were then further discussed and confirmed across the team.

## Results

**Fig. 1:**
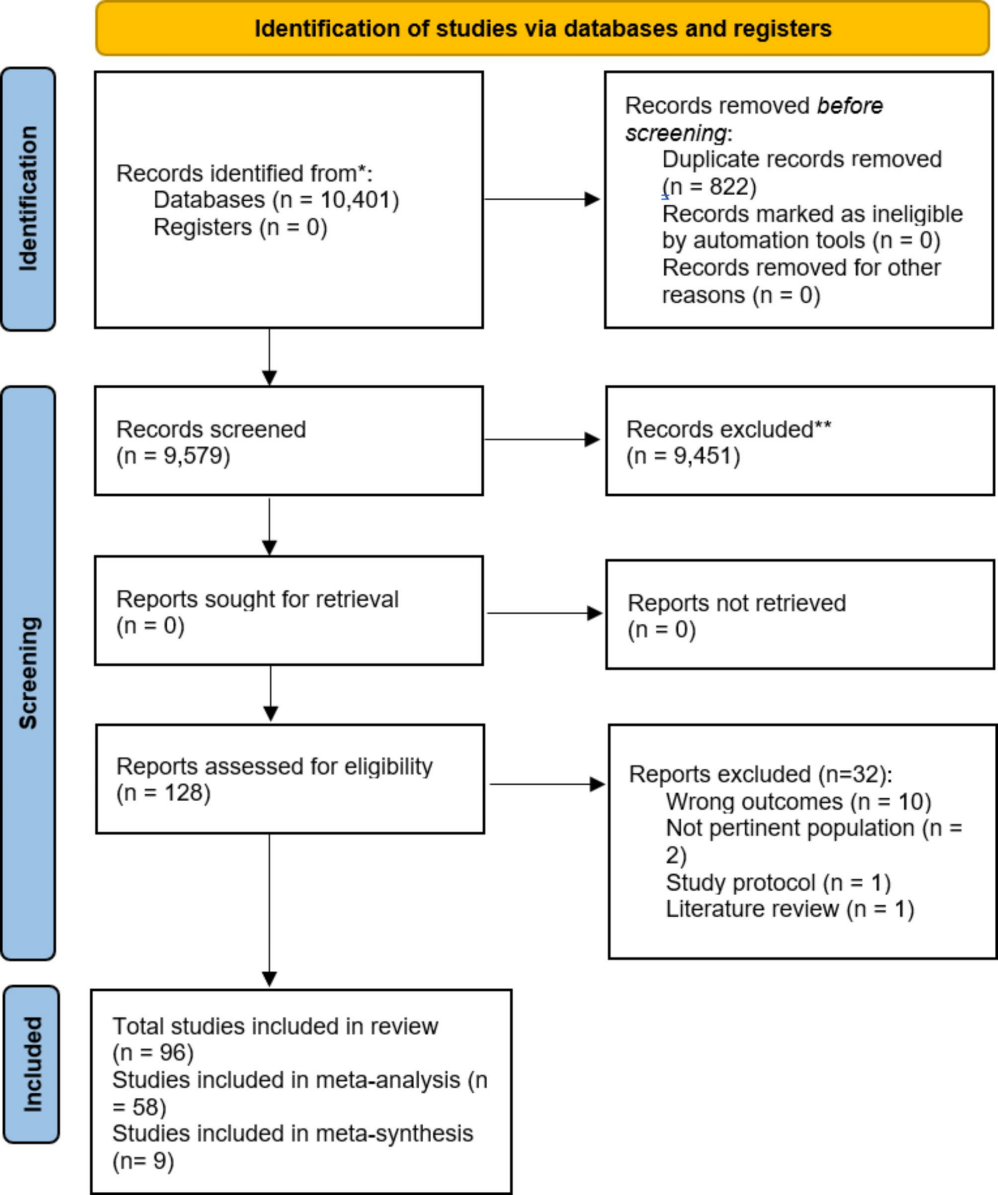
PRISMA diagram presenting process of selection and study screening

The database search initially identified 10,401 studies (including both quantitative and qualitative studies). Following duplicate removal, 9,579 studies remained. After screening titles and abstracts, 128 full text articles were subsequently reviewed. Of these, nine qualitative studies [19–27] were identified and included in the meta-synthesis (Fig. 1). No studies were excluded due to low-quality or unreliable research, with each study equally considered.

### Characteristics of studies

Of these nine studies [19–27], undertaken between 2015 and 2020, [23, 27] most were conducted in the UK, [20, 22, 23, 25] and in the USA, [19, 21, 27] with one study in Malaysia [24] and one study in Denmark. [26] All studies used either a qualitative [22–27] or mixed methods [19–21] approach. The studies focused on the following diseases: diabetes (n = 5), [20, 23–25, 27] hypertension (n = 3), [19, 21, 22] and COPD (n = 1). [26] Types of telemonitoring interventions used in each study varied (Table 1): four studies used participant’s mobile phones for data transmission, [19, 20, 22, 24] two studies provided tablet computers [26, 27] and two had home monitoring devices. [21, 23] One study recruited participants who had used/were using telemonitoring. [25] A usual care comparator was used by six studies, [20–24, 26] however only the telemonitoring intervention groups were interviewed. [20–24, 26] The remaining three studies were single groups with no comparator. [19, 25, 27] All studies collected data using semi-structured interviews (one study had an unspecified interview type), [22] either alone or using additional focus groups [21, 24, 27] or additional surveys. [19] Studies predominantly used thematic analysis [19, 20, 23, 24, 27] with one study each using grounded theory analysis, [23] Hamilton’s rapid analysis, [22] manifest and latent analysis [26] and phenomenological analysis. [25] Four studies focused only on patients’ views, [20, 24–26] whereas the remaining studies included patient, pharmacist, HCPs and carer views (see Table 1). Length of the intervention in included studies varied, with three [21, 22, 24] investigating telemonitoring use over 12 months, and five [20, 23, 26, 27] with interventions lasting between one and nine months. Characteristics of the population for each study is included in Table 1.

**Table 1:** Characteristics of the qualitative studies included in meta-synthesis

Author, Year Country	Participants Characteristics Sample size Age Gender Inclusion criteria	Methodology Study Design Analysis	Telemonitoring Intervention	Themes	Sub-themes
**Baron 2017** United Kingdom [20]	- 26 participants - Adults (age not specified) - Gender not specified for Qualitative data - English speaking Adults with T1DM/T2DM (HbA1c > 7.5%)	- Mixed methods study - Semi-structured interview. - Thematic Analysis	Mobile-phone based home telehealth (MTH) – transmitted diabetes related data (BG and BP readings, time since last meal, level of physical activity performed, insulin dose, weight) to MTH nurse for feedback.	1. Increased awareness 2. Increased motivation 3. Influence on diabetes self-care (reported to have increased the most in patients) 4. Perceived sense of security	1. - Level of diabetes control - Factors influencing diabetes control 2. - MTH is a motivational tool/personal challenge 3. - Increased monitoring of blood glucose - Dietary changes - Improved insulin intake and adjustments 4. - Someone there to monitor my clinical readings and prevent complications
**Beran 2018** United States [21]	- 27 participants – 23 patients, 4 pharmacists - Age not specified - Uncontrolled BP 140/90, Completed 54 month outcome research clinic	- Mixed methods study - Patient Focus Groups and Pharmacist semi-structured interviews - Grounded theory Analysis	Home BP monitors that stored and transmitted BP data to a secure website	1. Strong patient/pharmacist relationship 2. Individualised treatment plans 3. Communication among clinical staff (Insufficient communication with clinician) 4. frequent phone contact with pharmacist.	NA
**Buis 2020** United States [19]	- 15 participants − 13 patients, 1 pharmacist 1 physician **Patients**: - 18–65 - 53.3% Male, 46.7% Female - English speakers, possess smartphone compatible, uncontrolled HTN > 140/90 mmHg, under care at recruiting clinic taking at least 1 antihypertensive medication. **Stakeholders**: - Purposive sampling of individuals affiliated with BPTrack program or healthcare providers for enrolled patients	- Mixed methods study - Patient surveys and Patient interviews and Stakeholder semi-structured Interviews - Thematic Analysis	2 different mobile applications (one for patient, one for clinical pharmacist). Together allowed real-time electronic home blood pressure monitoring and medication adherence tracking.	1. Feasibility 2. Acceptability 3. Preliminary Effectiveness 4. Healthcare Utilisation	1. - Participant utilisation of BPTrack - Participant perceptions of Feasibility - Stakeholder Perceptions of Feasibility 2. - Participant perceptions of Acceptability - Stakeholder perceptions of acceptability 3. - Effect of BPTrack on Blood Pressure and Medication Adherence - Participant Perceptions of Effectiveness - Stakeholder Perceptions of Effectiveness
**Grant 2019** England [22]	- 23 patients, 2 carers, 15 Healthcare professionals - Patients, carers, and HCPs employed in practices based in West Midlands taking part in TASMINH4 RCT **Patients**: - > 35 - 78.2% male 21.7% Female - Uncontrolled HTN < 140/90 mmHg	- Qualitative Study, Interviews (type not stated) - Hamilton’s rapid analysis approach	Patients send BP readings via SMS text-based telemonitoring service. Patients alerted to contact their surgery in the light of very high or low readings, web page interpreted readings and graphically displayed BP readings.	1. Acceptability 2. Managing Data 3. Communication 4. Integrating self-monitoring in HTN management	N/A
**Hanley 2015** United Kingdom [23]	- 23 patients, purposively sampled, 10 professionals (4 GPs and 6 nurses) - Mean age 60 years (age of inclusion not included) - 70% Male, 30% Female - Inclusion criteria not included - At least 1 professional from each practice	- Qualitative study, Semi-structured interviews - Thematic analysis	Home BP, BG, weight monitoring - transmitted to a remote secure server which could be viewed by HCP and patient.	1. Contextual factors 2. Communication 3. Telemonitoring as support for managing the condition 4. The ‘fit’ of telemonitoring with personal lifestyles and professional practice	1. - Living with T2DM - Usual care - Preferred management options - Trialling 2. N/A 3. - Supporting self-care - Supporting treatment changes 4. N/A
**Lee 2018** England [25]	- 10 patients - > 18 - 49–77 - 80% Female, 20% Male - formal T2DM diagnosis, Received/receiving telehealth care for T2DM, - fluent English, able to provide informed consent for study	- Qualitative Semi-structured interviews - Phenomenological analysis	Recruited participants who have received or receiving telehealth care for T2DM	1. Technology consideration 2. Service perceptions 3. Empowerment	1. - Initial perception of using technology for self-management - Telehealth usability concerns 2. - Sense of security and comfort - Easy and convenience access to healthcare services - Privacy concerns - Continuity of care 3. - Patient education - Supporting self-care with telehealth system’s health trend analysis
**Lee 2019** Malaysia [24]	- 48 participants - 18–75 - 56% Females, 44% Males - Diagnosed with T2DM for at least 6 months (HbA1c 7.5-11%), regular access to internet, Randomised into intervention arms of IDEAS study - Non-probability sampling method	- Qualitative study - Semi-structured interview within focus groups - Thematic Analysis	Home monitoring of BG – transmitted to participant’s mobile phone to a remote secure server.	1. Generational differences 2. Independence and convenience 3. Sharing of health data and privacy 4. Concerns and challenges	N/A
**Nissen 2017** Denmark [26]	- 14 participants - 55–83 - 57.1% Female, 42.9% Male - selected from RCT net-COPD project group on a principle of maximum variation - Stable patients from outpatient clinic with severe and very severe COPD and at high risk of exacerbation	- Qualitative study, Semi-structured interviews - manifest and latent content analysis-	Tablet computer with web camera and microphone and measurement equipment (spirometer, pulse oximeter, scales). Readings submitted by patients to a call centre at patients local hospital and automatically categorised and prioritised. If red/yellow, patient would be contacted.	1. Sense of security and control 2. Knowing your disease 3. Virtues of the virtual consultation	1. - Keeping track - The lifeline 2. N/A 3. N/A
**Pekmezaris 2020** United States [27]	Pilot study Patients: - 12 participants - Age/gender not specified - Latin-X/Hispanic - T2DM receiving care form outpatient clinics in the New York Metropolitan area Community Advisory Board Members - 23 participants - H/L patients with T2DM, non-professional caregivers, disparity experts, clinicians, patient advocates and payor and health policy representatives	- Qualitative study - Focus groups and Semi structured interviews - Thematic Analysis	Tablet which provides patient with: (1) basic daily vital signs monitoring and facilitates nurse recognition of high BG. (2) weekly telemonitoring face-to-face video chat between patient and nurse. (3) culturally congruent educational videos concerning their condition	**Community advisory board and stakeholder focus groups** 1. Technology acceptance 2. Tablet interface 3. Video review 4. Consent Process concerns **Feedback from pilot study participants** **Adaptations implemented as a result of stakeholder feedback** 1. Changed to the patient-facing tablet screens 2. Changes to study procedures	1. N/A 2. Theatre testing: - Presentation of the information - Language use - Irrelevant information Community Advisory Board focus group: - Screen or verbiage changes - Desire for more training on using tablet - Video feedback 3. - Repetition of information - Presentation of information - Language choice - Cultural incongruence - Personal connection with actors 4. N/A

*BG = blood glucose, BP = blood pressure, COPD = Chronic obstructive pulmonary disease, H/L = Hispanic/Latino, HCP = healthcare professional, HTN = hypertension, MTH = mobile telehealth, RCT = randomised control trial, T1DM = Type 1 diabetes mellitus, T2DM = Type 2 diabetes mellitus*

### Quality assessment results

**Fig. 2:**
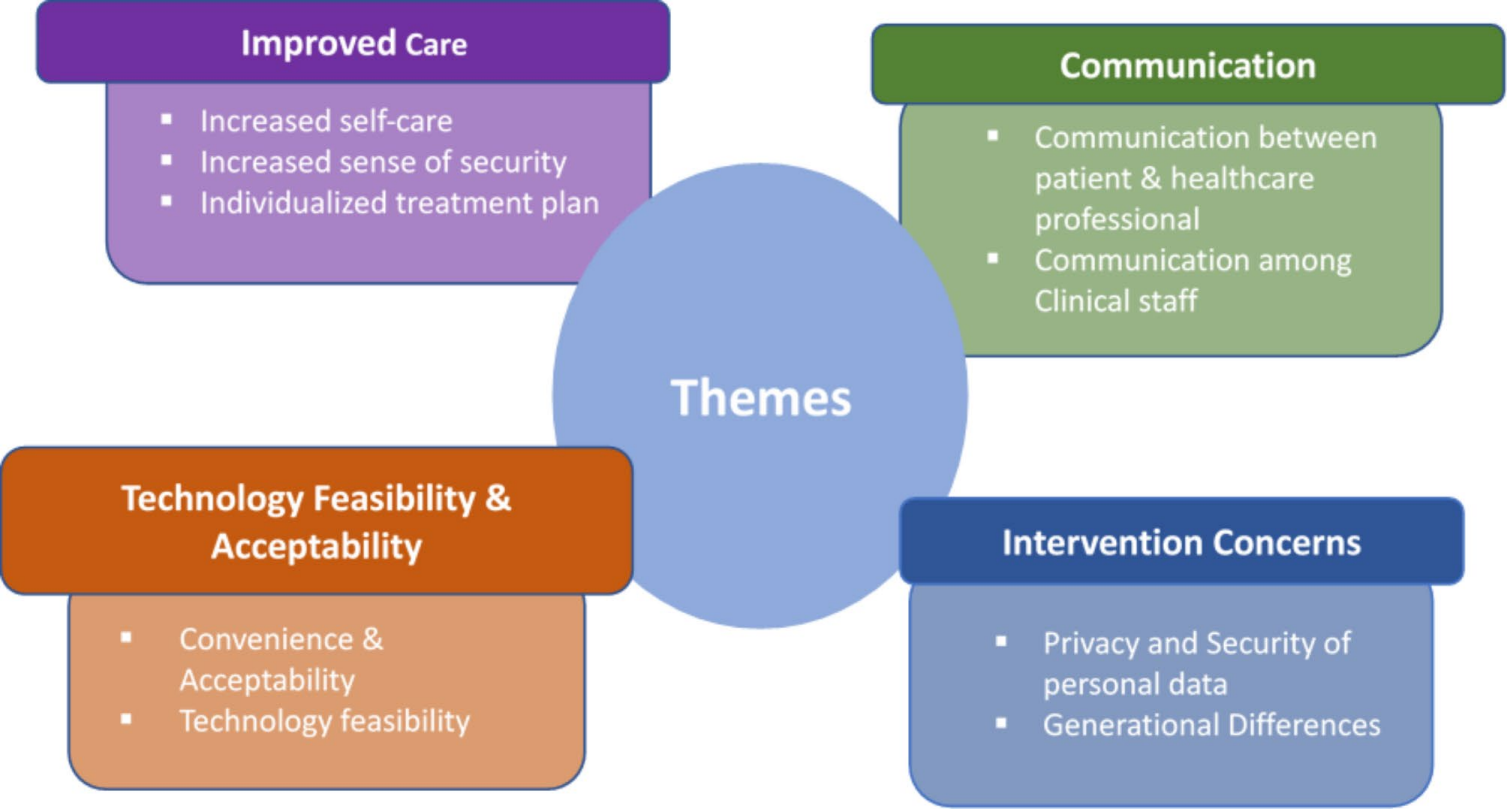
Summary of themes and subthemes in meta-synthesis

All studies [19–27] were deemed valid after being critically appraised (Table 2). However, insufficient details concerning patient-researcher relationship was seen in all nine studies, [19–27] resulting in the choice of ‘can’t tell’ being selected for this area. Three studies [19, 20, 27] were assessed as ‘can’t tell’ regarding use of an appropriate recruitment strategy. It was unclear in one study [20] how many participants from the randomised control trial agreed to be interviewed and how these were recruited. In another study [19], it was also unclear how patients were selected. This study may have also introduced bias through recruiting via purposive sampling of people affiliated with the BPTrack program (a home-monitoring intervention making blood pressure data available to pharmacists for the management of hypertension). [19] The last study [27] provided no explanation on participant recruitment. Most studies [19–24, 26, 27] showed insufficient details on how the research was explained to participants, consequently these were marked as ‘can’t tell’ in assessing whether ethical standards were maintained. The findings of the nine studies were categorised into four main (recurring) themes and a total of nine associated sub-themes (Fig. 2). Subsequent quotes for each theme are listed in Table 3.

**Table 2:** Quality Assessment summary of included qualitative studies in meta-synthesis

	Section A: Are the results valid?						Section B: What are the results?			Section C: will the results help locally?
**STUDY**	Clear Statement of the aims?	Appropriate qualitative methodology?	Appropriate research design?	Appropriate recruitment strategy?	Appropriate data collection?	Appropriate consideration of the patient-researcher relationship?	Ethical issues taken in consideration?	Rigorous data analysis?	Clear statement of findings?	How valuable is the research?
Baron 2017b	Y	Y	Y	CAN’T TELL	Y	CAN’T TELL	CAN’T TELL	Y	Y	Additional research is needed to identify mediators of change in this population
Beran 2018	Y	Y	Y	Y	Y	CAN’T TELL	CAN’T TELL	Y	Y	Investigates the relationship patient-pharmacist and clearly define the key characteristics for the successful of the intervention
Buis 2020	Y	Y	Y	CAN’T TELL	Y	CAN’T TELL	CAN’T TELL	Y	Y	Confirms the feasibility and acceptability of the intervention for this population.
Grant 2019	Y	Y	Y	Y	Y	CAN’T TELL	CAN’T TELL	Y	Y	Gives additional insights on the role of telemonitoring in BP management, showing its cost-effectiveness, easiness, and simplicity for self-management.
Hanley 2015	Y	Y	Y	Y	Y	CAN’T TELL	CAN’T TELL	Y	Y	Shows the beneficial effects of telemonitoring on patients self-care motivation and behaviour.
Lee 2018	Y	Y	Y	Y	Y	CAN’T TELL	Y	Y	Y	The findings support the use of telemonitoring for the routine care of people with type 2 diabetes.
Lee 2019	Y	Y	Y	Y	Y	CAN’T TELL	CAN’T TELL	Y	Y	Identification of several perceived barriers that may limit the effectiveness of telemonitoring for this population.
Nissen 2017	Y	Y	Y	Y	Y	CAN’T TELL	CAN’T TELL	Y	Y	Gives additional insights on the experience of telemonitoring for COPD patients with different severity of the condition.
Pekmezaris 2020	Y	Y	Y	CAN’T TELL	Y	CAN’T TELL	CAN’T TELL	Y	Y	Identifies important adaptation for telemonitoring interventions in this population (Hispanic/Latino).

**Table 3:** Quotes according to themes and subthemes in meta-synthesis

Theme	Subthemes and quotes
Improved Care	**Increased self-care** • “I get quite annoyed with myself when I see the reading go up, so I start investigating what have I been doing that’s done that” [20] • “It created an awareness within me to really pay attention to my diet, my salt intake, the types of food and, and become more involved with my overall health” [19] • “the minute I see my blood sugar high I definitely don’t eat anything” [23] • “I have to eat the right thing; I have to do this and that. It gives you the motivation to do the right thing…” [25] • “I went on the Net and found out everything I could about COPD” [26] **Increased sense of security** • “This is my health. Somebody is helping me to do it.” [20] • “You know at least, if something happens, you know that somebody is monitoring” [20] • “They’re looking after me” [22] • “I’m less anxious because I know someone’s always there on the other end” [25] • “This gives a feeling of security, and when you feel secure, you don’t hyperventilate” [26] **Individualized treatment plan** Positive • “[The pharmacist] was able to make some adjustments, and we were able to delete some medication and increase some medication and get everything to a nice manageable level.” [19] • “They need that close monitoring. They need someone to ask questions to who can really take the time and listen to them and really work with them to modify the regimen that suits them best” [21] • “His treatment is going to change quicker than it would have done normally” [23] • ‘This made it possible to initiate relevant treatment quickly’ [26] • “I was getting monthly readings and so that’s more intense follow-up” [22] Negative • “it’s not really going to change what we’ll do… I’ll change the medication based on the next HbA1c result” [23] • “we’ll… start throwing too many meds without having an adequate follow-up.” [19]
Communication	**Communication between patient & healthcare professional** Positive • “the pharmacist could see and take the time to look at readings…it made the biggest difference in the whole world” [21] • “I think what helped most was the instant feedback they got” [21] • “I do think they feel a lot more confident in partnership with the clinician, so I think it’s actually enhanced the doctor–patient relationship” [22] • ‘They further reported that the close contact with their nurses led to more honest answers and made it generally easier to talk about their health.’ [26] Negative • “The only thing that I do get a little bit peeved about is sometimes when they don’t get back to me.” [25] • “Dr. [name] has only been in touch once” [23] **Communication among Clinical staff** • “There is always a lack of communication between different characters…” [25] • “The Telehealth team’s trying to do one thing, and the doctors and matrons are doing something totally different. That’s the problem. They’re not really working together” [25] • “the doctor was -he was saying ‘well, am I taking care of your blood pressure, or is the pharmacist?’ He wasn’t even aware that I was part of this study, which might be a little bit of a weakness” [21]
Technology Feasibility & Acceptability	**Convenience & Acceptability** Positive • “It’s fantastic. I love it. It’s just so easy to access it quick…it’s just really useful” [22] • “Telehealth is much easier. Whereas you’re phoning a doctor you’ve got to wait, God knows how long!” [25] • “it stops me having to physically go so often to the doctors’ surgery and I would have thought it would save a lot of time on the nursing/doctor side.” [23] • “It’s so much easier we do not have to come to the clinic and can stay at home.” [24] • “if patients are able to see what their readings are in the house and keeping an eye on their weight and looking after their blood pressure…inevitably they will have better controlled diabetes…the time will free up for us.” [23] • “It’s become more personal with this screen … I think it’s an assembly line when you are at the respiratory outpatient clinic” [26] Negative • “I don’t know that I could take my blood pressure 3 times… every morning, and 3 times every night, for the rest of my life…” [19] • “I may be moved and get upset and perhaps cry a little, and I haven’t done that yet in these video consultations. But I know I would if I was sitting opposite and talking with her” [26] **Technology feasibility** Positive • “I say it is so easy to do once you get into the swing of it” [25] • “For me it’s not difficult … just need to teach (me) that’s all.” [24] • “I had no problems with the program” [19] Negative • “You have to train people from the most basic components of it if we’re going to be successful” [27] • “The… phone would unsync from the blood pressure. (Um-hm) And it would take several tries. Even though it said it paired, it did not pair.” [19] • “it didn’t have any cultural connection, like for my mom.” [27] • “it seems like it was an English translation into Spanish as opposed to a transcultural” [27] • “I already know to take my blood pressure medicine at the same time every day. So the repetitiveness of it just got annoying.” [19] • “Some- times, it would flash all through the day for the same question, which I’ve already answered the first time around…” [25] • “I worry that if they have to type in their readings, that they’re gonna put… you know, wrong numbers.” [19] **Barriers of rural areas** • “…whether you live in a village where (I feel) it will be very difficult… because (in) certain villages you don’t have (Internet) line” [24]
Intervention Concerns	**Privacy and Security of personal data** • “The only thing is, potentially, the confidentiality, if you’re texting back on the phone, as to who could potentially read it…if you were wanting to make some changes … you shouldn’t be doing it over a text anyway.” [22] • “I don’t like all my details like that for everybody to be monitoring. You don’t know who is at the other side….”. [25] • “what I am afraid of is (that the use of telemonitoring may) sometimes (cause) conflicts because it feels like you are being monitored by others.” [24] **Generational Differences** • ‘younger participants were more inclined to learn and use new technologies**’** [24] • “It has to be very simplistic, especially for our older patients.” [19] • “there are a lot of not elderly but more retired people and perhaps less confident at using mobile phones and texting is perhaps a younger population.” [22] • “I’m old (and) I need to write (the results) down. Anyway, as long as someone shows me how to do it, I can do it.” [24]

## Theme 1: Improved Care

The “improved care” theme was identified in seven of nine studies. [19–23, 25, 26] Key sub-themes included: (1) Increased self-care, (2) Improved sense of security and (3) Individualised treatment plan (Fig. 2).

### Increased self-care

Several patients expressed that telemonitoring increased their awareness of their health and consequently they made improved lifestyle changes. [19, 20, 23, 25, 26] Dietary alterations/improvements were a common outcome among several studies. [19, 20, 23, 25] Numerous patients with diabetes sought to actively reduce dietary sugar intake when raised blood glucose levels were identified through the telemonitoring device. [20, 23, 25] Similarly, patients with hypertension reported an increased awareness of salt intake [19] following high blood pressure readings. The patient was made aware of their blood glucose or blood pressure level via the software of the telemonitoring device, allowing them to make self-adjustment to their diet, and also providing educational feedback in some instances. [19]

Three studies [12, 20, 26] mentioned an increased understanding of their disease through telemonitoring, resulting in wider self-care improvements including better insulin adherence, increased blood glucose monitoring [20] and improved oxygen saturation monitoring. [12, 26].

### Improved sense of security

Four studies identified an increased sense of security. [20, 22, 25, 26] with patients feeling more relaxed knowing telemonitoring was enabling increased monitoring of their health parameters. For some, largely COPD patients, [26] this sense of security was considered to consequentially improve general health and well-being.

### Individualised treatment plans

Patients and HCPs reported additional benefits in having more individualised treatment plans [19, 21–23, 26] resulting from telemonitoring interventions. Several pharmacists observed making more responsive medication changes and individually tailored treatment plans through better-informed decisions. [21] One GP explained that receiving monthly digital readings resulted in a “*more intense (patient) follow-up*”. [22].

Whilst five studies [19, 21–23, 26] identified clear benefits of telemonitoring, some HCPs highlighted potential drawbacks concerning treatments. One pharmacist described a potential danger of prescribing additional medication without frequent face-to-face follow-ups. [19] Another GP stated that telemonitoring interventions would not alter medication administration. [23] However, in direct contrast, a nurse participating in the same study felt the telemonitoring intervention led to faster treatment changes; [23] a view shared by the participants of another included study. [26].

## Theme 2: communication

“Communication” emerged as a theme in five studies. [21–23, 25, 26] Associated sub-themes were: (1) Communication between patient & healthcare professional; and (2) Communication amongst clinical staff (Fig. 2).

### Communication between patient & healthcare professionals

One study [21] with a pharmacist as the patient’s main point of contact, indicated initial scepticism by patients over concerns regarding pharmacists’ qualifications. Nonetheless, most patients found frequent pharmacist contact and monitoring *“supportive and helpful*”. [21] The result of “*instant feedback*” and “*communication back and forth*” improved patient-professional relationships [21] with patients able to ‘open-up’ more regarding their health issues. [26] GPs reported improved communications resulting from telemonitoring interventions leading to *“enhanced doctor-patient relationships*”. [22] However, this view was inconsistent across studies; for example, some patients found communication difficult [25] or received minimal patient/HCP contact. [23] Some patients were also unaware who assessed the readings or if readings were being reviewed. [23].

### Communication amongst clinical staff

Some studies in which doctors were not the main contact points reported a “*lack of communication between staff*’’, [25] with patients repeating themselves to different staff members, resulting in the perception that HCPs were “*not working together*” with those monitoring the readings. [25] In one study patients complained that doctors were unaware about their participation in the study. [21].

## Theme 3: technology acceptability & feasibility

This theme arose in six studies [19, 22–25, 27] and comprise the following sub-themes: (1) Convenience & Acceptability; (2) Technology feasibility; and (3) Barriers of rural areas.

### Convenience & acceptability

Some participants (both HCPs and patients) thought using telemonitoring was “fantastic”, [22] finding it easy and quick to access, particularly in comparison to the difficulties of phoning doctors. [25] Patients commonly reported enjoying not having to attend surgery so often whilst being more regularly monitored. [23, 24] Participants considered that time-saving was a benefit for both HCPs and patients. [23] However, some patients were unhappy with having to self-monitor so often and were unsure how it would be maintained in the longer-term. [19] Some contrasting views were evidenced in one study concerning the personal element of virtual consultations. [26] In this study some individuals felt video consultations were safer and more relaxed than clinics and avoided the travel required to access the clinic in person). The importance of having the same nurse to interact with them was also highlighted, which provided a more familiar point of contact. However, some other participants did not feel comfortable talking about the psychological aspects of their disease via video-call, as they felt this approach was less “personal”, and they also reported distress when the internet signal was poor or interrupted during the call.

### Technology feasibility

Mixed opinions were evidenced concerning device utility. Whilst this is likely to be dependent on specific devices used, the importance of education regarding device usage was highlighted in three studies. [24, 25, 27] In one study, [19] multiple participants found difficulties in syncing blood pressure results to their phone, whilst others had “*no problems with the (telemedicine) programme*”. Two patients from other studies [19, 23] also had to be withdrawn following difficulties with equipment.

One study, [27] utilised a model for “modifying evidence-based interventions”, which provided several steps throughout the study allowing for participant feedback. Issues that emerged concerned technology types being used (e.g., tablet-based). Additionally, changes to devices were also suggested; changes to the screen, icons and verbiage used, for example suggesting changing words such as ‘podiatrist’ to ‘foot doctor’. In two other studies, [19, 25] patients recommended additional device changes, which included allowing customised messages [19] and limitations on daily notifications, as several patients complained about the devices’ high notification frequency. [19, 25] Consideration of cultural differences was highlighted when creating educational videos to supplement the device/application, as many patients felt the content and/or technology were not “culturally appropriate”, particularly targeting the Hispanic and Latino population [27].

HCPs also recommended new functionality be added – in which digital readings directly transferred from the device (‘BPTrack’) to the electronic health record. [19] Concerns over data input error were highlighted due to patients manually typing in readings, with the implication that devices able to automate data transfer might be better. [19].

### Barriers of rural areas

Differences between urban and rural areas in the acceptance of the technology were not exhaustively investigated in the included studies. However, participants in one study [24] were concerned about poor internet connectivity in certain villages, suggesting the results regarding technology feasibility may be dependent on the available digital infrastructure, thereby limiting its usability.

## Theme 4: intervention concerns

Concerns about the telemonitoring intervention were commonly reported [19, 22, 24, 25] across two sub-themes: (1) Privacy and security of personal data; and (2) Generational differences.

### Privacy and security of personal data

Some patients and HCPs expressed concerns over data confidentiality. [22, 24, 25] One GP thought this was of particular concern when using text messages as part of telemonitoring interventions. [22] One patient expressed discontent with not knowing who could access their personal details [25] which was supported by study participants with similar concerns in another study. [24].

### Generational differences

Participants within three studies [19, 22, 24] found demographic barriers in telemonitoring take-up. Older participants typically found telemonitoring devices more difficult to use than younger patients. [24] A physician described that self-monitoring programs needed to be “*very simplistic, especially for older patients*”. [19] Some HCPs were concerned that underpinning device technologies are inappropriate for the target age-groups. [22] Nonetheless, one older patient contradicted these views, explaining that “*as long as someone shows me… I can do it.”*, [24] suggesting that improvement to device education and training is likely to directly correlate with telemonitoring take-up.

## Discussion

This meta-synthesis addressed perceptions and experiences of telemonitoring users, highlighting barriers and facilitators resulting from increased implementation of telemonitoring. Four key themes were identified: Improved Care, Communication, Technology Acceptability, and Feasibility and Intervention Concerns. Results from this meta-synthesis suggest that telemonitoring is both feasible and acceptable for chronic health condition management. Most participants had positive experiences from telemonitoring usage, with most reporting that it is more convenient than attending in-person clinics. Many HCPs and patients supported this view, believing telemonitoring saved healthcare time/resource and improved communication. Frequent contact and monitoring from telemonitoring increased patient satisfaction due to a greater perceived sense of security. Patients’ perceptions were further improved by individually focused treatment plans. Many patients also reported increased motivation to improve/manage their lifestyles through improvements to daily health measures. This, supplemented with educational videos, gave patients the necessary information to make these lifestyle changes, which may lead to better patient health outcomes.

None of the included studies investigated telemonitoring use over a long timeframe. Only one study [24] recruited patients with previous telemonitoring experience of 1.5–3.5 years; however, the sample size was only 10, with no information specifying the exact length of experience participants had within this range. Of the remaining studies, length of the intervention was between one and 12 months, making therefore difficult to assess whether patients would remain permanently engaged and motivated to self-manage their disease(s) over a sustained period of time.

Barriers were also identified regarding the technologies being used. In three studies, [19, 23, 27] participants found devices difficult to use, which was accentuated by generational differences, with older participants generally less confident and less able to use them. Three studies [24, 25, 27] however, suggested this barrier could be better managed with improved education and support. Personal data security was also a concern amongst several participants, suggesting that further safeguards to improve privacy and data security of telemonitoring devices (or education about security) are beneficial as part of wider clinical implementation.

### Comparison with previous research

Novel findings from this meta-synthesis highlight the importance of providing sufficient device education and support which may help to overcome barriers surrounding acceptability. It has also drawn attention to concerns regarding security of personal data, not seen in other meta-syntheses. [12–14] Few meta-syntheses [12–14] are available that focus on telemonitoring within chronic disease management. Our review is unique in assessing more than one chronic disease across adults of all ages, and our findings show consistency with other reviews on telemonitoring. [12, 13] Indeed, our findings align with two other studies reporting that telemonitoring is likely to improve patients’ self-care [28] and sense of security. [12] A study using a digital health intervention for management of depression, anxiety, and somatoform disorders [13] also highlighted convenience benefits associated with telemonitoring (e.g., reducing stigma to access treatment, improve self-management of the condition). Another meta-synthesis identified telemonitoring uptake being constrained by various technological issues associated with equipment usage. [12] Several patients stopped using telemonitoring altogether for technological barriers (e.g., difficulty in using the telehealth device – especially in older population), a finding also in line with previous literature. [12].

This review is accompanied by a complimentary systematic review [15] and meta-analysis. When compared to usual care, patient adherence was improved in telemonitoring groups in six of twelve studies assessed. Patient satisfaction was also assessed in nine studies, with five concluding that patient satisfaction was improved by telemonitoring. These studies, however, were not included in the meta-analysis due to variation in patient satisfaction definition(s). It is considered beneficial to additionally assess patient satisfaction using a qualitative approach to gather further information on telemonitoring facilitators and barriers. Whilst quality of life was found to not be significantly improved in the systematic review, this meta-synthesis provides additional evidence on how patient satisfaction and life-quality can be improved through telemonitoring usage.

Strengths and Limitations.

The inclusion criteria of this meta-synthesis encompass more than one chronic disease and had few exclusion criteria, unlike previous meta-syntheses. Studies reporting the use of different devices were also included as well as perspectives from both HCPs and patients, providing more expansive results. The results may therefore be more generalizable and provide stronger evidence for implications for clinical practice. Rigorous quality assessment was employed, with two reviewers independently screening for inclusion/exclusion criteria and independently assessing for risk of bias with an appropriate tool.

Nevertheless, several limitations are worth noting. Identified studies reported barriers and facilitators of the whole intervention, but did not provide enough information on the individual components of the intervention that may have acted as barrier/facilitator. However, the information gathered was able to provide a good overview of the difficulties encountered by the participants that used telemonitoring, as well as identifying points for improvement to be considered for future telemonitoring interventions. Furthermore, the low number of studies included in this meta-synthesis may affect the generalizability of the findings’, particularly as included studies were mostly conducted in the UK and USA. The low number of studies and the wide variety of interventions presented (which ranged from telephone monitoring to mobile apps), may have impacted the results of this meta-synthesis, as patients’ perception of telemonitoring (such as “easiness of use” of the devices) may have been affected. Despite conducting a comprehensive and systematic database search, the low number of identified papers suggests that there is limited research that has assessed the experience of using telemonitoring using qualitative methodology. No grey literature search was conducted, which may have led to some relevant studies being omitted.

Implication for clinical practice and future studies.

Our findings suggest that telemonitoring may be a useful tool to support clinical practice, which may lead to improved patient satisfaction and care. The main barriers to increased implementation of telemonitoring primarily relate to device usage (e.g., difficulties in using the device). Further research is necessary to assess the benefits of different device technologies in addressing long-term condition management and patient engagement. Appropriate education and tailoring of the intervention (e.g., type of technology used, frequency of the measurements, intensity of the support) based on the patients’ needs/requirements may be required. In addition, attention should be given in building user-friendly interfaces for these devices that can reduce potential barriers to utlising this technology (especially for older patients). Further research may also be required to determine the most appropriate clinical staff member(s) to act as the main point(s) of contact for telemonitoring interventions.

## Conclusion

This meta-synthesis of telemonitoring user perceptions in managing chronic disease suggests that telemonitoring is both feasible and acceptable. Most participants preferred the convenience of telemonitoring in comparison to attending in-person clinics and took comfort from increased communication with HCPs. Many patients increased efforts in managing their lifestyle with the motivation of improving daily health measurements. Personal data security however remains an ongoing concern for some users, which must be addressed before more permanent implementation in clinical practice. Additional barriers to telemonitoring uptake include an ongoing unfamiliarity with technologies being used, particularly for older people. Further targeted research is therefore required to identify the most effective telemonitoring technologies across a broad demographic, and to ensure appropriate support and training in the use of the technology is provided.

### Electronic supplementary material

Below is the link to the electronic supplementary material.

Supplementary Material 1

## Data Availability

All data generated or analysed during this study are included in this published article [and its supplementary information files].
