# Supplementary material for: Use of telemonitoring in patient self-management of chronic disease: a qualitative meta-synthesis
Source: BMC Cardiovasc Disord. 2023 Sep 19;23:469. doi: 10.1186/s12872-023-03486-3 (PMC10510185; doi:10.1186/s12872-023-03486-3)
Supplement: Supplementary file 1 — Supplementary Material 1 [file 12872_2023_3486_MOESM1_ESM.docx]

**Supplementary Table 1**: Search Strategy for Ovid MEDLINE

| **Terms** | **Results** |
| --- | --- |
| #1.exp Telemedicine/ | 32,271 |
| #2.exp Telecommunications/ | 97,220 |
| #3.Telecare.mp. | 788 |
| #4.Telehealth.mp. | 6,616 |
| #5.e-technology.mp. | 64 |
| #6.mobile health.mp. | 8,718 |
| #7.interactive.mp. | 56,578 |
| #.8 remote monitoring.mp. | 2,518 |
| #.9 automated alert.mp. | 63 |
| #10.exp Videoconferencing/ | 2,123 |
| #11.digital.mp. | 144,370 |
| #12.exp Self-Management/ | 2,781 |
| #13.exp Telephone/ | 23,302 |
| #14.exp Smartphone/ | 5,212 |
| #15.exp Cell Phone/ | 11.244 |
| #16.anroid.mp. | 2,798 |
| #17.iphone.mp. | 910 |
| #18.IOS.mp. | 1,727 |
| #19.device.mp. | 292,723 |
| #20.mobile device.mp. | 1,292 |
| #21.store-and-forward.mp. | 584 |
| #22.e-health.mp. | 3,067 |
| #23.m-health.mp. | 629 |
| #24.exp Cardiovascular Disease/ | 2,430,366 |
| #25.exp Heart Diseases/ | 1,148,108 |
| #26.exp Heart Failure/ | 125,042 |
| #27.cardiac surgery.mp. | 43,106 |
| #28.exp Thoracic Surgery/ | 12,822 |
| #29.exp Myocardial Infarction/ | 178,034 |
| #30.exp Myocardial Ischemia/ | 436,437 |
| #31.silent myocardial infarction.mp. | 267 |
| #32.silent myocardial ischemia.mp. | 1,162 |
| #33. Exp Coronary Artery Disease/ | 64,329 |
| #34. Exp Angina pectoris/ | 43,665 |
| #35. Exp Stroke/ | 139,798 |
| #36. Exp Pacemaker, Artificial/ | 27,673 |
| #37. Exp Defibrillators/ | 18,722 |
| #38. Exp Defibrillators. Implantable/ or ICD.mp | 47,712 |
| #39. Exp Atrial Fibrillation/ or AF.mp. | 93,862 |
| #40. Afib.mp. | 350 |
| #41. copd.mp. or exp Pulmonary Disease, Chronic Obstructive/ | 75,878 |
| #42.exp Diabetes Mellitus/ | 435,708 |
| #43. Chronic disease.mp or exp Chronic Disease/ | 294,090 |
| #44.exp Hypertension/ | 258,047 |
| #45. Exp Arrhythmias, Cardiac/ | 213,352 |
| #46. #24 or #25 or #26 or #27 or #28 or #29 or #30 or #31 or #32 or #33 or #34 or #35 or #36 or #37 or #38 or #39 or #40 or #41 or #42 or #43 or #44 or #45 | 3,148,500 |
| #47. #1 or #2 or #3 or #4 or #5 or #6 or #7 or #8 or #9 or #10 or #11 or #12 or #13 or #14 or #15 or #16 or #17 or #18 or #19 or #20 or #21 or #22 or #23 | 589,041 |
| #48. #46 and #47 | 88,113 |
| #49 limit #48 to (humans and last 11 years) | 48.017 |
| #50 (randomized controlled trials or controlled clinical trial or pragmatic clinical trial or multicentre study).pt or non-randomized controlled trials as topic/ or interrupted time series analysis/ or controlled before-after- studies/ or (ramdomis* or randomiz* or randomly).ti,ab. or trial.ti. or (before adj5 after).mp. or (pre adj5 post).mp. or ((pretest or pre test) and (posttest or post test)).mp. or quasiexperiment*.mp. or quasi experiment*.mp or time series.mp. or repeated measure*.ti.,ab. | 1,875,395 |
| #51. #49 and #50 | 10,401 |
